# Associations of education and income with heavy drinking and problem drinking among men: evidence from a population-based study in Japan

**DOI:** 10.1186/s12889-019-6790-5

**Published:** 2019-04-23

**Authors:** Keiko Murakami, Hideki Hashimoto

**Affiliations:** 1 Tohoku Medical Megabank Organization, Tohoku University, 2-1 Seiryo-machi, Aoba-ku, Sendai, Miyagi 980-8573 Japan; 2 Graduate School of Medicine, Tohoku University, 2-1 Seiryo-machi, Aoba-ku, Sendai, Miyagi 980-8573 Japan; 3 Department of Health and Social Behavior, School of Public Health, The University of Tokyo, 7-3-1 Hongo, Bunkyo-ku, Tokyo, 113-0033 Japan

**Keywords:** Education, Income, Japan, Heavy drinking, Problem drinking, General population

## Abstract

**Background:**

Some studies in Western countries have suggested that education and income are differentially associated with different drinking patterns. This study aimed to examine the associations of education and income with heavy drinking and problem drinking among community-dwelling Japanese men.

**Methods:**

A questionnaire survey was conducted in metropolitan areas in Japan from 2010 to 2011 among residents aged 25 to 50 years; valid responses were received from 2004 men. Drinking patterns were categorized as non-to-moderate drinking, non-problematic heavy drinking, and problem drinking. Multiple logistic regression analyses were conducted to determine whether educational attainment or income was associated with drinking patterns, after adjustment for age, marital status, working status, income/education, self-rated health, and psychological distress.

**Results:**

The study population included 84.4% non-to-moderate drinkers, 8.9% non-problematic heavy drinkers, and 6.7% problem drinkers. Lower educational attainment (high school or less) was significantly associated with increased risks of both non-problematic heavy drinking (odds ratio [OR], 1.80; 95% confidence interval [CI], 1.21–2.67) and problem drinking (OR, 2.06; 95% CI, 1.34–3.16), compared with university education or higher. Lower income (lowest tertile) was significantly associated with a lower risk of non-problematic heavy drinking (OR, 0.66; 95% CI, 0.43–1.00), but not of problem drinking (OR, 0.80; 95% CI, 0.50–1.30), compared with the highest income tertile.

**Conclusions:**

These findings indicate that education and income are differentially associated with alcohol drinking patterns among community-dwelling Japanese men.

## Background

Harmful alcohol use is one of the world’s leading risk factors for morbidity, disability, and mortality. Approximately 3.3 million deaths (5.9% of all global deaths) and 139 million disability-adjusted life years (5.1% of the global burden of disease and injury) are attributable to alcohol use [1]. Therefore, preventing and reducing harmful alcohol use is a public health priority. To design appropriate public health policies, it is important to understand the population groups that are most affected by harmful alcohol use.

Alcohol-attributable health harm generally tends to be more prevalent in lower social strata, which is particularly the case for men [2]. Drinking patterns, at least in part, may help account for this differential burden of harm [2]. Although the literature overwhelmingly indicates that those with lower socioeconomic status (SES) are more likely than others to have unhealthy lifestyles with respect to smoking, diet, and exercise [3], the evidence is inconsistent in the case of alcohol use [4, 5].

It is possible that some of the inconsistent findings may be explained by different social and cultural contexts across countries and variations in the assessment of SES and drinking patterns. National differences in the degree of inequality in alcohol use between different SES groups may result from the fact that social patterns of drinking are largely the result of cultural and environmental influences and of government policies in the countries concerned [6]. Evidence on socioeconomic differences in drinking patterns have been reported mainly from Western countries; these differences may look different in Japan, where there is a relatively high alcohol tolerance and men attend regular after-work drinking meetings with work colleagues [7]. Some studies in Western countries have suggested that education and income are differentially associated with drinking patterns; the propensity to engage in hazardous drinking is greater for less-educated men, and men with higher income tend to consume more alcohol and more frequently than those who are less affluent [8]. For example, high income earners are more likely to be frequent drinkers, presumably because they can afford to purchase alcoholic beverages and have more social opportunities that include alcohol consumption. Those with low educational attainment are more likely to be binge drinkers because of more frequent exposure to social stress and low health literacy regarding the health hazards of alcohol use [9]. Education and income capture distinct aspects in society, though these reflect a central dimension of social stratification [10, 11]. To our knowledge, the associations between SES and heavy drinking have not been examined using both education and income as SES indicators among community-dwelling Japanese men. In addition, although a failure to account for the multidimensionality of drinking patterns may explain some of the inconsistency in findings across previous studies [12–14], no studies in Japan have examined heavy drinking and alcohol-related problems simultaneously.

The aim of the present study was to examine the associations of education and income with heavy drinking and problem drinking among community-dwelling Japanese men.

## Methods

### Study population

**Fig. 1:**
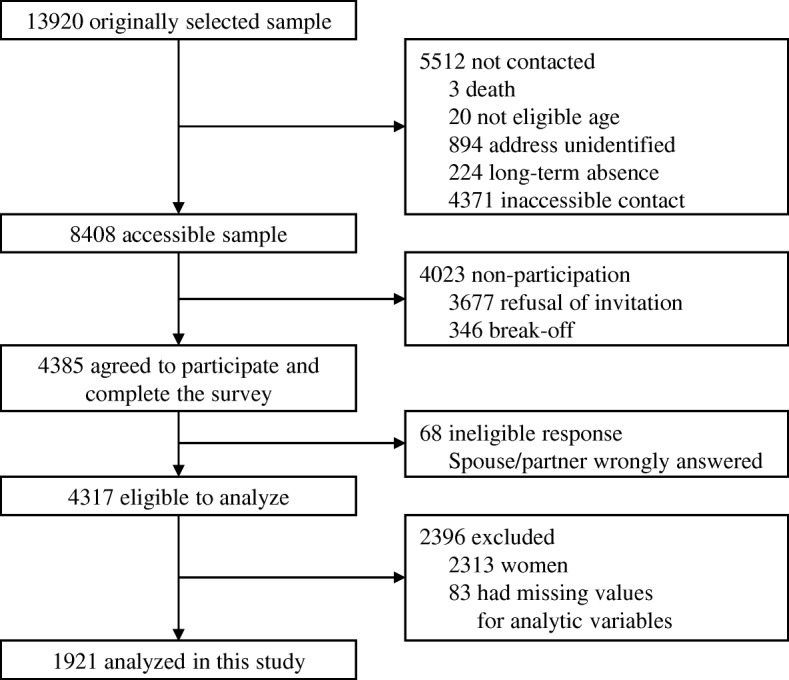
Flow diagram of participants in the present analysis of the Japanese Study on Stratification, Health, Income, and Neighborhood (J-SHINE)

Data were obtained from the Japanese Study of Stratification, Health, Income, and Neighborhood (J-SHINE), which has been described elsewhere [15, 16]. The survey was conducted in four municipalities in and around the greater Tokyo metropolitan area between July 2010 and February 2011. Among 13920 adults aged 25 to 50 years who were probabilistically selected from the residential registry in each of these four municipalities, survey staff members were able to contact 8408 residents. Valid responses were received from 4317 residents, 2004 of whom were men. We analyzed the responses of 1921 men who had no missing values on the variables used in the analysis, excluding income. Figure 1 shows the flow diagram of the present study.

### Educational attainment and equivalent income

Participants were sorted into three categories according to educational attainment: high school or lower (elementary, junior high school, or senior high school), college (2-year college or special training school), and university or higher (university or graduate school). We calculated equivalent income as household income adjusted for household size, using the OECD-modified equivalence scale [17]. For participants whose household income was missing or unknown but who responded their individual income, individual income was used as equivalent income. Missing values after this step were imputed using a single imputation based on regression analysis including age, marital status, working status, self-rated health, psychological distress, and educational attainment.

### Alcohol drinking patterns

The frequency of alcohol consumption in the past year was classified into the following six categories: every day, 5–6 days/week, 3–4 days/week, 1–2 days/week, a few times/month, or seldom/can’t. Current drinkers (≥ a few times/month) were asked to report the types of drinks consumed and their average consumption per day: beer, *shochu* (white spirits), *sake* (rice wine), whisky, wine, and/or *shochu* highball. We assigned a score to each category of alcohol consumption frequency as follows: 7 for every day, 5.5 for 5–6 days/week, 3.5 for 3–4 days/week, 1.5 for 1–2 days/week, and 0.5 for a few times/month. The ethanol equivalent intake in grams was calculated as follows: 633 ml beer or *shochu* highball = 23 g of ethanol, 180 ml *shochu* = 38 g, 180 ml *sake* = 23 g, 60 ml whisky = 23 g, and 120 ml wine = 11.5 g. Finally, weekly ethanol equivalent intake was estimated by multiplying the amount of ethanol by the frequency score; daily ethanol equivalent intake was calculated by dividing these estimates by seven. Participants who did not consume alcohol in the past year were considered abstainers and were assigned 0 g/day.

Several Japanese studies have demonstrated that ≥2 ‘*gou’* of per day increases the risk of lifestyle-related disease for men [18, 19]. In Japan, 1 ‘*gou’* is equivalent to approximately 180 ml of *sake*, or 23 g of ethanol, and is the most common unit for measuring the amount of alcohol consumed. Heavy drinking for men in the present study was therefore defined as ethanol intake ≥46 g/day (≥2 *gou*/day).

All participants who consumed alcohol ≥2 *gou*/day were asked to complete the CAGE questionnaire [20, 21]. CAGE is an acronym based on the following four questions: Have you ever felt you ought to *cut down* on your drinking? Have people *annoyed* you by criticizing your drinking? Have you ever felt bad or *guilty* about your drinking? Have you ever had a drink first thing in the morning to steady your nerves or get rid of a hangover (*eye-opener*)? These items are used to create a simple drinking problem scale, with each positive response given a score of 1; a higher score indicates the presence of an alcohol problem. While the CAGE does not provide standard Diagnostic and Statistical Manual diagnosis of alcohol dependence, a positive response on two or more questions indicates a high likelihood of the presence of problematic drinking [20, 21]. Problem drinking was therefore defined in the present study as a CAGE score of 2–4.

### Covariates

We selected the following covariates: age, marital status (married/common-law, others), working status (working, not working), self-rated health [22, 23], and psychological distress [22, 24]. Self-rated health was assessed with one question “How would you describe your health? Would you say it is excellent, very good, good, fair, or poor?” Responses were dichotomized as good health (excellent, very good, or good) and poor health (fair or poor) for purposes of analysis. Psychological distress was assessed with the Japanese version of the K6 scale, which consists of six items assessing depressive moods and anxiety in the past 30 days. Responses range from 0 (none of the time) to 4 (all of the time), with total scores ranging from 0 to 24 [25, 26]. Participants were dichotomized into those with psychological distress (a total score of the K6 scale ≥5) and those without psychological distress (0–4 score) [26, 27].

### Statistical analysis

We compared characteristics of participants with analysis of variance for continuous variables and the chi-squared test for categorical variables. Drinking patterns were categorized as non-to-moderate drinking (< 46 g/day of ethanol intake), non-problematic heavy drinking (≥46 g/day of ethanol intake and CAGE score of 0 or 1), and problem drinking (≥46 g/day of ethanol intake and CAGE score of 2–4). We conducted multiple logistic regression analyses to examine the associations of education and income with non-problematic heavy drinking and problem drinking. For each outcome, we calculated the odds ratios (ORs) and 95% confidence intervals (CIs) for education or income adjusted for age (model 1a, 1b), as well as for marital status, working status, and income/education (model 2). We made further adjustments for self-rated health and psychological distress (model 3). Non-drinkers were also included in the reference group along with moderate drinkers, in accordance with previous Japanese studies [8, 28].

All analyses were conducted with Stata 14.0 (StataCorp LP, College Station, TX, USA). For all analyses, a two-tailed *P* value < 0.05 was considered statistically significant.

## Results

Table 1 shows the characteristics of participants. In the study population, 1621 participants (84.4%) were non-to-moderate drinkers, 171 (8.9%) were non-problematic heavy drinkers, and 129 (6.7%) were problem drinkers. More than half of the participants had graduated from university or higher; mean age was 37.3 years (standard deviation, 7.2 years). Non-problematic heavy drinkers were older, more likely to be married by formal or common law, more likely to be currently working, and less likely to rate their own health as poor or have psychological distress than non-to-moderate drinkers and problem drinkers.

**Table 1:** Characteristics of participants: the Japanese Study on Stratification, Health, Income, and Neighborhood (J-SHINE)

	Total (*N* = 1921)		Drinking patterns						*P*-value^a^
			Non-to-moderate drinking (*n* = 1621)		Non-problematic heavy drinking (*n* = 171)		Problem drinking(*n* = 129)		
Educational attainment, n (%)									< 0.001
University or higher	1041	(54.2)	910	(56.1)	78	(45.6)	53	(41.1)	
College	419	(21.8)	348	(21.5)	42	(24.6)	29	(22.5)	
High school or lower	461	(24.0)	363	(22.4)	51	(29.8)	47	(36.4)	
Equivalent income^b^, mean (SD)	3839.8	(2142.8)	3781.4	(2112.9)	4306.1	(2167.2)	3956.5	(2405.7)	0.008
Age, mean (SD)	37.3	(7.2)	36.7	(7.1)	41.3	(6.3)	39.6	(7.4)	< 0.001
Married/common-law, n (%)	1276	(66.4)	1041	(64.2)	135	(79.0)	100	(77.5)	< 0.001
Working, n (%)	1788	(93.1)	1498	(92.4)	166	(97.1)	124	(96.1)	0.027
Poor self-rated health, n (%)	203	(10.6)	168	(10.4)	12	(7.0)	23	(17.8)	0.008
Psychological distress, n (%)	705	(36.7)	600	(37.0)	39	(22.8)	66	(51.2)	< 0.001

*Non-to-moderate drinking*: < 46 g/day of ethanol intake; *Non-problematic heavy drinking*: ≥46 g/day of ethanol intake and CAGE score of 0 or 1; *Problem drinking*: ≥46 g/day of ethanol intake and CAGE score of 2–4

^a^Obtained using analysis of variance for continuous variables and the chi-square test for categorical variables, comparing drinking patterns

^b^Thousand Japanese yen (/year)

Table 2 presents the ORs and 95% CIs for non-problematic heavy drinking compared with non-to-moderate drinking. Lower educational attainment was significantly associated with an increased risk of non-problematic heavy drinking after adjusting for age, marital status, working status, and income (model 2), as well as for self-rated health and psychological distress (model 3); the multivariate-adjusted ORs of high school education or lower compared with university education or higher were 1.73 (95% CI, 1.17–2.57) and 1.80 (95% CI, 1.21–2.67), respectively. Lower equivalent income was significantly associated with a lower risk of non-problematic heavy drinking after adjusting for age, marital status, working status, and education (model 2), as well as for self-rated health and psychological distress (model 3); the multivariate-adjusted ORs of the lowest compared with highest income tertile was 0.63 (95% CI, 0.42–0.96) and 0.66 (95% CI, 0.43–1.00), respectively.

**Table 2:** Odds ratio (OR) and 95% confidence interval (CI) for non-problematic heavy drinking compared with non-to-moderate drinking

	Model 1a	Model 1b	Model 2	Model 3
	OR (95% CI)	OR (95% CI)	OR (95% CI)	OR (95% CI)
Educational attainment				
University or higher	1.00		1.00	1.00
College	1.55 (1.03–2.32)		1.76 (1.16–2.68)	1.79 (1.17–2.72)
High school or lower	1.55 (1.06–2.27)		1.73 (1.17–2.57)	1.80 (1.21–2.67)
Equivalent income				
3rd tertile (highest)		1.00	1.00	1.00
2nd tertile		0.86 (0.58–1.28)	0.75 (0.50–1.12)	0.77 (0.52–1.16)
1st tertile (lowest)		0.73 (0.50–1.08)	0.63 (0.42–0.96)	0.66 (0.43–1.00)
Covariates				
Age	1.11 (1.08–1.13)	1.10 (1.07–1.13)	1.09 (1.06–1.12)	1.09 (1.06–1.12)
Marital status				
Married/common-law			1.00	1.00
Others			0.75 (0.50–1.13)	0.80 (0.53–1.22)
Working status				
Working			1.00	1.00
Not working			0.62 (0.24–1.63)	0.74 (0.28–1.96)
Self-rated health				
Good				1.00
Poor				0.82 (0.43–1.56)
Psychological distress				
No				1.00
Yes				0.61 (0.41–0.90)

*Non-to-moderate drinking*: < 46 g/day of ethanol intake; *Non-problematic heavy drinking*: ≥46 g/day of ethanol intake and CAGE score of 0 or 1

Model 1a, 1b: adjusted for age

Model 2: model 1 + adjusted for marital status, working status, and equivalent income/educational attainment

Model 3: model 2 + adjusted for self-rated health and psychological distress

Table 3 presents the ORs and 95% CIs for problem drinking compared with non-to-moderate drinking. Lower educational attainment was significantly associated with an increased risk of problem drinking after adjusting for age, marital status, working status, and income (model 2), as well as for self-rated health and psychological distress (model 3); the multivariate-adjusted ORs of high school education or lower compared with university education or higher were 2.17 (95% CI, 1.41–3.32) and 2.06 (95% CI, 1.34–3.16), respectively. Lower equivalent income was not associated with problem drinking after adjusting for age, marital status, working status, and education (model 2), as well as for self-rated health and psychological distress (model 3); the multivariate-adjusted ORs of the lowest compared with highest income tertile were 0.86 (95% CI, 0.54–1.40) and 0.80 (95% CI, 0.50–1.30), respectively.

**Table 3:** Odds ratio (OR) and 95% confidence interval (CI) for problem drinking compared with non-to-moderate drinking

	Model 1a	Model 1b	Model 2	Model 3
	OR (95% CI)	OR (95% CI)	OR (95% CI)	OR (95% CI)
Educational attainment				
University or higher	1.00		1.00	1.00
College	1.47 (0.92–2.36)		1.52 (0.93–2.46)	1.47 (0.90–2.39)
High school or lower	2.13 (1.41–3.22)		2.17 (1.41–3.32)	2.06 (1.34–3.16)
Equivalent income				
3rd tertile (highest)		1.00	1.00	1.00
2nd tertile		1.32 (0.84–2.06)	1.11 (0.70–1.77)	1.06 (0.67–1.70)
1st tertile (lowest)		1.04 (0.66–1.63)	0.86 (0.54–1.40)	0.80 (0.50–1.30)
Covariates				
Age	1.06 (1.03–1.09)	1.06 (1.03–1.09)	1.05 (1.02–1.08)	1.05 (1.02–1.08)
Marital status				
Married/common-law			1.00	1.00
Others			0.72 (0.45–1.14)	0.63 (0.40–1.01)
Working status				
Working			1.00	1.00
Not working			0.75 (0.28–1.98)	0.54 (0.20–1.46)
Self-rated health				
Good				1.00
Poor				1.65 (0.98–2.76)
Psychological distress				
No				1.00
Yes				1.95 (1.33–2.86)

*Non-to-moderate drinking*: < 46 g/day of ethanol intake; *Problem drinking*: ≥46 g/day of ethanol intake and CAGE score of 2–4

Model 1a, 1b: adjusted for age

Model 2: model 1 + adjusted for marital status, working status, and equivalent income/educational attainment

Model 3: model 2 + adjusted for self-rated health and psychological distress

Psychological distress was significantly associated with a lower risk of non-problematic heavy drinking and an increased risk of problem drinking. Similar associations were observed for self-rated health, although these associations were not significant.

## Discussion

We examined the associations of education and income with alcohol drinking patterns among community-dwelling Japanese men. Men with lower education had significantly higher risks of both non-problematic heavy drinking and problem drinking. In contrast, men with lower income had a lower risk of non-problematic heavy drinking, while income was not associated with problem drinking.

Lower education was significantly associated with increased risks of both non-problematic heavy drinking and problem drinking; this result was consistent with previous findings [4, 29]. Education conveys factual health-related knowledge and raises cognitive skills that affect health-promoting decisions [10, 30, 31]. Hence, education may increase individual’s understanding of the negative effects of heavy drinking and may build individual’s capacity to manage drinking by stopping or keeping consumption low [9, 32]. Education also shapes cultural capital [33] in the form of health-related values and norms [34, 35]. Because alcohol drinking is influenced by cultural norms that are relatively straightforward [8], unequal distribution of cultural capital across educational levels may result in differences in alcohol drinking patterns. Social networks, which combine individual’s resources with those of others [36], may also partially explain education-related inequalities in heavy drinking. Given that those with higher education adopt health-promoting behaviors and associate with others with higher education, their social networks communicate health-promoting behaviors and widen education-related inequalities [3, 31, 36]. Drinking patterns can follow social networking paths [37], and therefore cement education-related inequalities in heavy drinking.

For non-problematic heavy drinking, distinct associations with education and income were observed: those with lower education had a significantly higher risk, while those with higher income were also more likely to be non-problematic heavy drinkers. Most studies in Japan have evaluated either education or income and have shown somewhat inconsistent results [28, 38–42]. International comparisons of drinking patterns according to SES reported by the OECD indicated that men in Japan who were less educated were more likely to be heavy drinkers [8]. In contrast, in 2014 the National Health and Nutrition Survey, which consists of a nationally representative sample in Japan, found that higher household income was associated with an increased risk of heavy drinking among men [28]. Using both education and income as SES indicators, we confirmed that education and income were differentially associated with heavy drinking.

We found that high income was significantly associated with an increased risk of non-problematic heavy drinking. Evidence on the associations between income and drinking patterns is somewhat unclear; some studies found that heavy drinking was more prevalent among those with higher income [2], whereas others found that higher income was associated with a higher frequency of light drinking [9, 43]. Comparisons at the national level have reported little correlation between per capita purchasing power parity-adjusted GDP and adult consumption of alcohol or alcohol abstention rates among richer countries [2]. One possible explanation for the finding that higher income was associated with a higher risk of non-problematic heavy drinking in the present study is that those with higher income have more disposable income with which to purchase alcohol [44]. However, because safe and high-quality varieties of nearly every kind of alcoholic beverage are available at relatively low prices in Japan [45], this explanation seems insufficient. Another explanation is that unlike smoking, which is generally perceived as unacceptable, drinking is often an integral part of social life, especially in the working environments where those with higher income operate. In Japan, drinking is an important social event, especially among middle-aged men; individual drinking patterns might reflect the opportunities for social drinking [46]. In the Japanese cultural context, men are work-oriented and may spend several nights a week socializing with work colleagues after work in “drinking meetings,” which are lubricated by copious amounts of alcohol [7]. The availability of enough money to purchase alcoholic beverages and work-related networking that accelerates social drinking can explain the association between high income and heavy drinking.

In contrast, equivalent income was not associated with problem drinking, defined as alcohol dependence and alcohol abuse as well as having alcohol-related problems. Some studies have demonstrated that the association between SES and drinking patterns was relatively larger for more extreme drinking behavior [14, 47], while others showed that SES had similar associations with problem drinking and heavy drinking [29, 32, 48]. The different associations in the present study between income and non-problematic heavy drinking versus problem drinking suggest that these drinking patterns reflect different characteristics and have different determinants in Japan.

We also found different directions of association between psychological distress and non-problematic heavy drinking versus problem drinking. Our findings suggest that those who are psychologically positive tend to drink heavily but without problems at social drinking events, whereas those with psychological problems tend to drink to the point of problem drinking to reduce these problems [49]; however, the causal relationship remains unclear. The association of education and income with drinking patterns did not change substantially even after adjusting for psychological distress and self-rated health, indicating that psychological distress and self-rated health cannot fully explain these associations.

These findings have some implications for alcohol drinking policy. The present study showed that those with less education are more likely to report both non-problematic heavy drinking and problem drinking. Although the group that reported these behaviors was small, it may have a large impact on the social distribution of disease burden and health service use. It would therefore be beneficial to improve knowledge and literacy regarding the health hazards of alcohol use, as well as cultural capital and social networks enhancing health-promoting behaviors. In contrast, the lack of an association between income and problem drinking suggests that taxation and pricing policies may not be successful in reducing problem drinking. It would instead be beneficial to treat psychological distress and to increase knowledge and literacy to reduce problem drinking. Most preventive measures concerning alcohol-related harm reduction target society as a whole; comparatively little is known about effective measures to target those with low SES, and therefore new approaches are required [2].

The present study has several limitations. First, the response rate was low. If non-respondents had lower SES than did respondents as mentioned in some previous studies, socioeconomic inequalities in drinking patterns may have been underestimated. However, the participants of the J-SHINE survey were comparable with the vital statistics of the target population in terms of age, sex, and educational attainment [15]. Second, the sampled municipalities were all located in urban areas, where demographics and social norms regarding alcohol drinking may be different from those in rural areas. The findings should therefore be generalized only with caution. Third, as in most large-scale studies, we relied on self-reported alcohol consumption. However, one study showed that correcting self-reporting bias resulted in minimal change in the direction and magnitude of education-related differences in heavy drinking among Japanese men [50]. Fourth, we examined current drinking rather than drinking history, and therefore did not distinguish between lifetime abstainers and former drinkers, possibly affecting the interpretation if former drinkers were those who stopped drinking because of ill health. Finally, due to the cross-sectional nature of the study, the causal direction of the associations observed was not determined. Though adulthood drinking patterns may have little influence on educational attainment, it is possible that these patterns affect current income because heavy drinking and problem drinking have harmful effects on physical and mental health, which may cause loss of earnings or unemployment.

## Conclusions

We examined the associations of education and income with heavy drinking and problem drinking among community-dwelling Japanese men. Men with lower education had significantly higher risks of both non-problematic heavy drinking and problem drinking. In contrast, men with lower income had a lower risk of non-problematic heavy drinking, while income was not associated with problem drinking. These findings imply that education and income are differentially associated with alcohol drinking patterns; these associations are important in designing interventions to reduce alcohol drinking inequalities in alcohol drinking among community-dwelling Japanese men.

## Abbreviations

95% CI: 95% confidence interval
J-SHINE: Japanese Study of Stratification, Health, Income, and Neighborhood
OR: Odds ratio
SES: Socioeconomic status
